# Climate change impacts on aflatoxin B_1_ in maize and aflatoxin M_1_ in milk: A case study of maize grown in Eastern Europe and imported to the Netherlands

**DOI:** 10.1371/journal.pone.0218956

**Published:** 2019-06-27

**Authors:** H. J. Van der Fels-Klerx, L. C. Vermeulen, A. K. Gavai, C. Liu

**Affiliations:** Wageningen Food Safety Research (previously RIKILT), Wageningen University and Research, Wageningen, the Netherlands; University of Illinois, UNITED STATES

## Abstract

Various models and datasets related to aflatoxins in the maize and dairy production chain have been developed and used but they have not yet been linked with each other. This study aimed to investigate the impacts of climate change on aflatoxin B_1_ production in maize and its consequences on aflatoxin M_1_ contamination in dairy cow’s milk, using a full chain modelling approach. To this end, available models and input data were chained together in a modelling framework. As a case study, we focused on maize grown in Eastern Europe and imported to the Netherlands to be fed–as part of dairy cows’ compound feed–to dairy cows in the Netherlands. Three different climate models, one aflatoxin B_1_ prediction model and five different carryover models were used. For this particular case study of East European maize, most of the calculations suggest an increase (up to 50%) of maximum mean aflatoxin M_1_ in milk by 2030, except for one climate (DMI) model suggesting a decrease. Results from all combinations of carryover and climate models suggest a similar or slight increase (up to 0.6%) of the chance of finding aflatoxin M_1_ in milk above the EC limit of 0.05 μg/kg by 2030. Results varied mainly with the climate model data and carryover model considered. The model framework infrastructure is flexible so that forecasting models for other mycotoxins or other food safety hazards as well as other production chains, together with necessary input databases, can easily be included as well. This modelling framework for the first time links datasets and models related to aflatoxin B_1_ in maize and related aflatoxin M_1_ the dairy production chain to obtain a unique predictive methodology based on Monte Carlo simulation. Such an integrated approach with scenario analysis provides possibilities for policy makers and risk managers to study the effects of changes in the beginning of the chain on the end product.

## Introduction

Aflatoxins are a group of related mycotoxins. Mycotoxins are toxic compounds produced as secondary metabolites by several toxigenic fungi upon and after infection of crops. Given their occurrence and toxicity, the European Commission (EC) has set limits for the combined presence of the four aflatoxins B_1_, B_2_, G_1_, and G_2_, as well as for the presence of aflatoxin B_1_ (AfB1) in feed and aflatoxin M_1_ (AfM1) in food [1, 2]. Aflatoxins B_2_, B_2a_, G_1_, G_2_ and G_2a_ as well as M_1_ appear to be conversion products of AfB1 [3]. Aflatoxins were classified by the International Agency for Research on Cancer (IARC) as class 1, implying “there is sufficient evidence in humans for the carcinogenicity of aflatoxins” [4]. AfB1 is mainly produced by *Aspergillus flavus* and *A*. *parasiticus* in feed and food crops, such as maize [5]. When AfB1 occurs in feed and is consumed by dairy cattle, a variety of symptoms can occur, which includes un-thriftiness, anorexia and decreased milk production [3]. The digestion system of the lactating animals (e.g. dairy cows, goats and sheep) converts some of the ingested AfB1 into AfM1 which is excreted in milk [3, 6]. As a result, AfM1 can be present in dairy products, such as milk, leading to exposure of humans to this toxic component. Since milk is an important foodstuff for adults and children, the presence of AfM1 can pose a risk to human health [7]. In Europe, a tolerable daily intake of AfM1 has been set at 0.2 ng/kg b.w. and limits have been set for its maximum presence in food products, i.e., 0.05μg/kg for raw milk, heat-treated milk and milk for the manufacture of milk-based products [1]. In 2013, a large shipment of maize from East Europe, which was distributed to feed producers in Germany and the Netherlands, was found to be contaminated with AfB1. AfB1 concentrations, as reported by Germany to the Rapid Alert System Food and Feed (RASFF) system, ranged from 21–204 μg/kg, which were far above the EC legal limit for AfB1 in maize for feed (20 μg/kg). Dairy farms in Germany and the Netherlands were affected as well, since AfM1 was found in the milk at farms that used compound feed produced from the AfB1 contaminated maize, and a major recall started. As a result of this incident, the Dutch feed industry has largely intensified their regular monitoring of AfB1 in maize intended for feed production [8]. Additionally, the Netherlands Food and Consumer Product Safety Authority also monitors the presence of AfB1 in maize used for feed production, as part of the National Plan Animal Feed. Infection of the host crop (in this case maize) by the responsible fungus *A*. *flavus* is to a large extent influenced by local weather and by agronomical practices, such as the cultivar, tillage method, and crop rotation [9, 10]. Weather conditions facilitating infection of the host crop with *A*. *flavus* are warm and humid conditions as well as droughts. Temperature, precipitation patterns and other climate factors are expected to change due to greenhouse gas emissions. Climate modelling studies project that temperature will continue to increase gradually over time, resulting in a 2 °C to 5 °C increase of 1-in-20 year extreme daily maximum temperature by the late 21^st^ century [11]. The distribution of precipitation is expected to change, resulting in an increase in the number of extreme precipitation events [12]. In many mid-latitude dry regions (i.e. maize growing area), mean precipitation will likely decrease, while in many mid-latitude wet regions, mean precipitation will likely increase [13]. These changes will likely affect infection of maize with *A*. *flavus* and resulting AfB1 contamination. A previous study has estimated that within the next 100 years, a 2 °C or 5 °C increase in temperature is expected to increase AfB1 contamination of maize in parts of Europe, especially with the +2 °C scenario, relative to their baseline of 1975–2005 [9, 14, 15]. It should be noted that scenario analysis is a tool to explore different plausible futures, but does not give information about the probability of occurrence of a given outcome [16].

So far, parts of the dairy supply chain have been modelled: the effect of climate change on AfB1 contamination of maize; prediction of AfB1 contamination of maize (ingredient of compound feed to dairy cows), and; carryover from AfB1 intake by dairy cows into AfM1 in milk produced by these cows [6, 9, 10, 14, 17]. To date, the various datasets and models related to AfB1 in the maize and AfM1 in the dairy production chain have not yet been linked with each other. If such an integrated approach would be taken, the effects of changes in the beginning of the chain on the final product, the cow’s milk, could be estimated. Such scenario analyses could be done with the aim of facilitating decision making by policy makers and other risk managers.

The aim of the current study was to investigate the impacts of climate change on aflatoxin B_1_ production in maize and its consequences on aflatoxin M_1_ contamination in dairy cow’s milk, using a full chain modelling approach. Based on the 2013 incident described above, we focused on milk production on a typical Dutch dairy farm that uses compound feed for the dairy cows with imported maize from Eastern Europe (Ukraine) as an ingredient, as a case study to demonstrate the potential of chain modelling and scenario analysis for food safety.

## Material and methods

A modelling approach was used in which data and available models at various stages of the dairy production chain, from maize cultivation to cow’s milk production, were linked with each other. For our case study, we constrained our models based on a specific grain (Maize), animal (Cow), toxin (Aflatoxin), and country (Netherlands). We focused the modelling of the dairy production chain in the Netherlands, which currently imports most of its maize from Eastern Europe, in particular from Ukraine [18]. Consequently, we performed the maize growing scenario analysis for maize grown in Ukraine. The general setup of the model chain, input data, and details of the various models are explained in the following sections. All data and models are available (S1 File).

### Model linking

**Fig 1:**
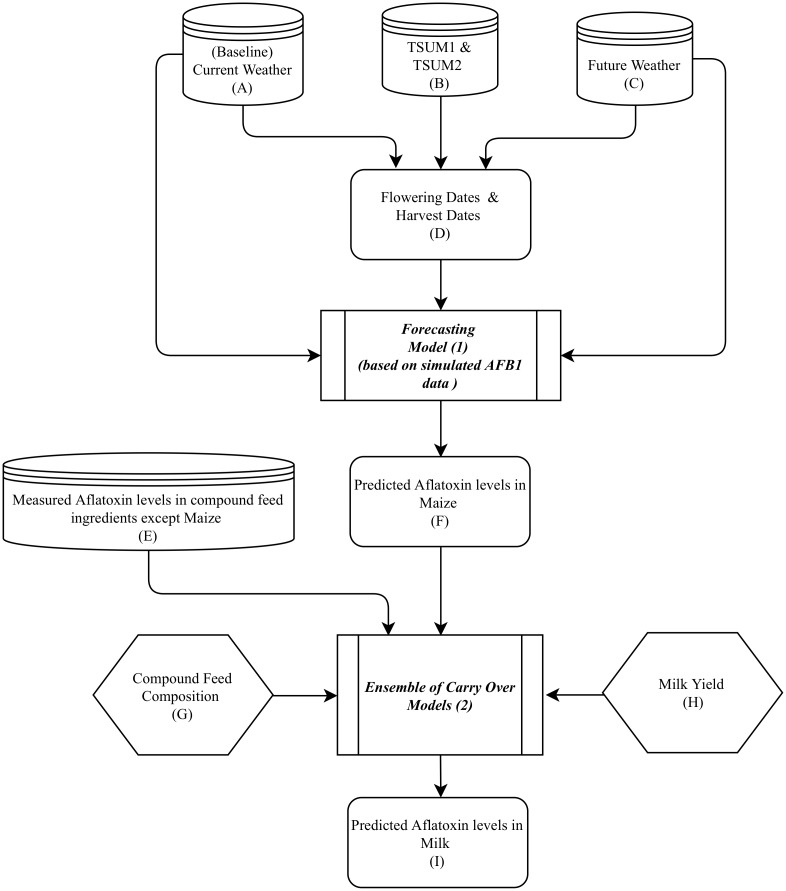
Set-up of the model chain. Box 1–2 show the two models that are linked. Box A-D provide the input for the forecasting model, and box E-H are the input for the Carryover model. Box F and I are predicted model outcomes.

A Forecasting Model [10, 17] and a Carryover Model [6] were used in our setup (Fig 1). The general setup is a sequence of data and models that are chained together, and in which output from one model forms the basis for input to another model (Fig 1). Maize flowering and harvest dates (D, Fig 1) were estimated using temperature sum (TSUM) data (B, Fig1) from the Joint Research Centre (JRC) crop database and weather data from the JRC weather database (C, Fig 1). The Forecasting model (1, Fig 1) predicts AfB1 concentrations in maize based on simulating fungal dispersal, growth and AfB1 production, using the estimated flowering and harvest dates (D, Fig 1) and data on the model weather variables (A, C, Fig 1) as input. Weather data and TSUM data from Ukraine were used as a case study to run the Forecasting model. Input data covered all European countries’ baseline weather data and 90 years of simulated future weather representing the year 2030 produced by three climate models (European Commission Joint Research Centre (JRC) agri4cast.jrc.ec.europa.eu/DataPortal/), see section Data Used for details on input data. Then, for data of the baseline and the three climate models, we determined the mean and standard deviation of the predicted AfB1 contamination levels in maize grown (F, Fig 1) in Ukraine now and in the future. These were used as input for contamination of maize used as compound feed in the Carryover model (2, Fig 1), that predicts consequent AfM1 concentrations in milk (I, Fig 1) produced in the Netherlands. Calculations of AfM1 concentrations in milk were performed for the case of the bulk milk production of a typical Dutch dairy farm with 69 cows. The Carryover model used all transfer equations that are currently available (published in scientific literature) to account for uncertainty and variability in the transfer of AfB1 in dairy cows’ feed to AfM1 in dairy cows’ milk. The Carryover model was run 10000 times using Monte Carlo sampling. Section Models Used provides more details on the models.

In principle, this model chain approach is easily scalable when local parameters for the Carryover model (e.g., compound feed composition and milk yield) are known. The Forecasting model could be run for maize grown in all countries in Europe, as weather data are available for all countries from the JRC database.

### Data used

#### Crop phenology data

Data on emergence date, temperature sum (TSUM) per grid cell (25km × 25km) from the crop phenology model WOFOST (WOrld FOod STudies) developed under EU Monitoring Agricultural ResourceS (MARS) project were used to estimate flowering dates and harvest dates of maize [19]. TSUM is a fixed value, per grid, of temperature sum to estimate flowering date and harvest date relative to emergence date. TSUM1 is the accumulated daily average temperature from emergence date until flowering date. TSUM2 is the accumulated daily average temperature from flowering date to harvest date. Gridded TSUM1 and TSUM2 values, as available in the JRC database, were used to estimate flowering date (TSUM1) and harvest date (TSUM1 + TSUM2) of the maize crop for each grid cell. For maize, TSUM starts to accumulate when daily average temperature is between 6°C and 30°C. The temperature out of this range is considered not suitable for maize to grow [19].

#### Weather data

Input data in the forecasting model includes gridded weather data from the European Commission Joint Research Centre (JRC). These data contains meteorological variables from weather stations interpolated on a 25km × 25km grid and are available via the Agri4Cast Data Portal (agri4cast.jrc.ec.europa.eu/DataPortal/). The variables maximum air temperature (°C), minimum air temperature (°C), mean air temperature (°C), sum of precipitation (mm/day) and vapour pressure (hPa) were used in the Forecasting model. Relative humidity (%) was calculated from vapour pressure and saturation vapour pressure based on Tetens equation [20]:

$$RH=\frac{VP}{es}\times 100$$ (1)

Where, VP is vapour pressure (converted to kPa) and es is the saturation vapour pressure (kPa).

$$es=6.1078\times e^{\frac{17.269\times T}{237.3+T}}$$ (2)

Where, es is saturation vapour pressure in kPa, T is temperature in °C. As baseline weather data the years 2005–2017 were used [21]. This period was chosen so as to have the most recent data, but sufficient years to even out potential anomalies.

#### Climate scenario data

A database of future daily weather data is available for the whole of Europe, specifically designed to be used for crop modelling [22]. In short, these data are based on three downscaled and bias-corrected regional climate model implementations of the IPCC A1B emission scenario, which is a middle of road scenario describing a world with rapid economic growth and a mix of fossil and non-fossil energy sources, as created within the ENSEMBLES project [23–25]. The weather generator ClimGen [26] has been applied to produce 30 simulated years of weather for each climate model, for the time horizons 2000, 2020 and 2030. These future weather data are bias-corrected using the same weather database as our forecasting model baseline [21]. Duveiller et al. [22] term the three climate model implementations DMI-HIRHAM5-ECHAM5 [26], ETHZ-CLM-HadCM3Q0 [27] and METO-HC-HadRM3Q0-HadCM3Q0 [28], which are respectively referred to as DMI, ETHZ and METO for short in this paper. Duveiller et al. chose these models because they are based on widely used global circulation models and show maximum diversity in output weather variables. In general, DMI and METO are the coldest and warmest regarding surface air temperature, respectively, ETHZ falls in between but shows different precipitation patterns [22]. We uses the 2030 time horizon for all three climate models for our scenario analysis. For each climate model, 30 simulated years representing 2030 were used, meaning 90 years of potential future European weather in 2030. The data are available on a 25 x 25 km grid. A data summary is presented in Table 1.

**Table 1:** Summarized mean and standard deviation (SD) baseline climate data and projected changes for 2030 for each of the three climate models for Ukraine*.

Statistic	Baseline	2030		
		*DMI*	*ETHZ*	*METO*
**Mean Tmin (°C)**	10.4	10.6	10.9	11.4
**SD Tmin**	6.5	6.2	6.5	6.9
**Mean Tmax (°C)**	20.8	20.3	20.6	21.3
**SD Tmax**	8.8	8.2	8.6	9.0
**Mean sum of precipitation (mm/year)**	360	333	244	250
**SD sum of precipitation**	120	127	120	99

* According to the projections, Ukraine’s mean minimum temperature will increase according to all models. Mean maximum temperature will go either down (DMI, ETHZ) or up (METO). Total annual precipitation will decrease according to all models, most strongly with ETHZ.

### Models used

#### Forecasting model

The forecasting model used in the current modelling chain is a simplified version of the AFLA-maize model developed by Battilani et al. [10] and the aflatoxin simulation model by Chauhan et al. [17]. The AFLA-maize model [10] was used as the basis. The full model structure, parameter coefficients and validation are detailed in the original publication [10]. The global accuracy of AFLA-maize model is 68% [10]. AFLA-maize is a mechanistic model that simulates complexed fate of fungus and eventually their mycotoxin production. An Aflatoxin Risk Index (ARI) is calculated from silk emergence to crop harvest by the process of fungal sporulation, spore dispersal, germination, colony growth and aflatoxin production. Each step is an equation resulting from intensive experiments. These equations are calculated with inputs including temperature, precipitation, relative humidity, leaf wetness, available water, water activity or maize growth stage. In their aflatoxin simulation model, Chauhan et al. [17] assumed that *A*. *flavus* fungal inoculum was always available for infection and hence sporulation and germination were considered non-limiting factors in the forecasting model. Therefore, in this study, ARI was computed only with dispersal, growth and aflatoxin production processes. Aflatoxin concentrations were then estimated from ARI by a linear regression equation. A set of local data (anonymised AfB1 data published in FigShare) on AfB1 concentrations in maize were used to calculate the parameter coefficient. The following equation was obtained, which was consequently applied to translate ARI into AfB1 concentrations in the predictive model:

$$Aflatoxin=10^{0.047765\times ARI}-1$$ (3)

#### Carryover model

The aflatoxin Carryover simulation model [6] aims to estimate the concentrations of AfM1 in milk produced at the dairy farm from AfB1 intake of the dairy cows at the farm. The model simulates bulk milk production at a typical dairy farm in the Netherlands with an assumed average herd size of 69 dairy cows[6]. The most common breed in the Netherlands for milk production is the Holstein-Friesian bred. The influence of individual differences between cows (size and breed) were not considered in the original study because milk batch contamination is only relevant at the farm level. The individual difference between cows would smooth out in the results. This model therefore assumed an average total daily feed intake (and standard deviation) of 18.7 (1.3) kg DM (Dry Matter)/cow, of which 4.3 (0.2) kg DM/cow is compound feed [6]. The Carryover model can be run with six different scenarios for compound feed composition and milk production (relative to feed intake) of the cows. These six scenarios are the results of: 1) three compound feed composition scenarios, namely High-Protein, Low-Protein and Feed Ingredient with Minimal and Maximal ranges (i.e. 0%-35% maize in compound feed), and 2) two different milk yield scenarios, namely normal and extreme lactation [6]. For the current case, we used the compound feed composition scenario with Minimal and Maximal ranges and the normal milk yield scenario [6] to limit the number of scenarios for the demonstration purpose. With the Minimal and Maximal ranges, AfB1 concentrations in other feedstuffs than maize it taken from the ranges found in the national monitoring program in the Netherlands. The method presents in this study can be used to predict aflatoxin contamination under all six scenarios. Intake of roughage by dairy cows was not considered since roughage fed to dairy cows in the Netherlands (grass and silage) has a low probability to be contaminated with AfB1. The model is based on Monte Carlo simulation (10000 iterations), to account for variation and uncertainty related to the input variables of the model, mainly AfB1 concentrations in the feed ingredients and the carryover of AfB1 in maize to AfM1 in milk. The carryover of AfB1 from the feed–into the cow’s body–to AfM1 in the milk was simulated by using published carryover equations. The transfer rate used to set the EC legislative limit in feed for dairy cows was 1%-2% [6]. All five different carryover equations [29–33], used by Van der Fels-Klerx and Camenzuli [6], were implemented in the model approach covering current variation and uncertainty. These five equations are presented in Table 2.

**Table 2:** Equations used for modelling the transfer of AfB1 in feed to AfM1 in milk.

Equation	Source
${\left[AfM1\right]}_{milk}(\frac{{\mu g}_{AfM1}}{{kg}_{milk}})=\frac{{Total\;intake}_{AfB1}}{Y_{milk}}\times \frac{0.5154\times e^{Y_{milk}\times 0.0521}}{100}$	[29]
${\left[AfM1\right]}_{milk}(\frac{{ng}_{AfM1}}{{kg}_{milk}})={Total\;intake}_{AfB1}\times 0.787+10.95$	[30]
${\left[AfM1\right]}_{milk}(\frac{{\mu g}_{AfM1}}{{kg}_{milk}})=\frac{{Total\;intake}_{AfB1}}{Y_{milk}}\times \frac{Y_{milk}\times 0.077-0.326}{100}$	[31]
${\left[AfM1\right]}_{milk}(\frac{{\mu g}_{AfM1}}{{kg}_{milk}})={Total\;intake}_{AfB1}\times \frac{Y_{milk}\times 0.032}{17+Y_{milk}}$	[32]
${\left[AfM1\right]}_{milk}(\frac{{\mu g}_{AfM1}}{{kg}_{milk}})=\frac{{Total\;intake}_{AfB1}}{Y_{milk}}\times \frac{Y_{milk}\times 0.13-0.26}{100}$	[33]

The carryover model uses AfB1 intake of the cows as input. AfB1 intake was considered a distribution, resulting from the amount of compound feed intake per cow (fixed value), the compound feed composition (range for inclusion rate per ingredient) and the presence of AfB1 in the compound feed (distribution of AfB1 per ingredient). For the latter, data on AfB1 concentrations in the feed ingredients were obtained from a database with 10 years of national monitoring results. However, data on the AfB1 concentration in maize used for compound feed production for dairy cows were replaced by the estimated AfB1 concentrations in maize, resulting from the aflatoxin Forecasting model, either in the baseline or given climate change data as inputs. To do so, mean and standard deviation (SD) of the estimated AfB1 concentrations over all Ukrainian grids and years were calculated and used for the distribution of AfB1 in maize. Model output is weekly resolved and aggregated for the whole farm. Aflatoxin binders are not commonly used in compound feed production in the Netherlands and their effect was therefore not included.

## Results

### Predicted aflatoxin concentrations in Ukrainian maize under climate change

**Fig 2:**
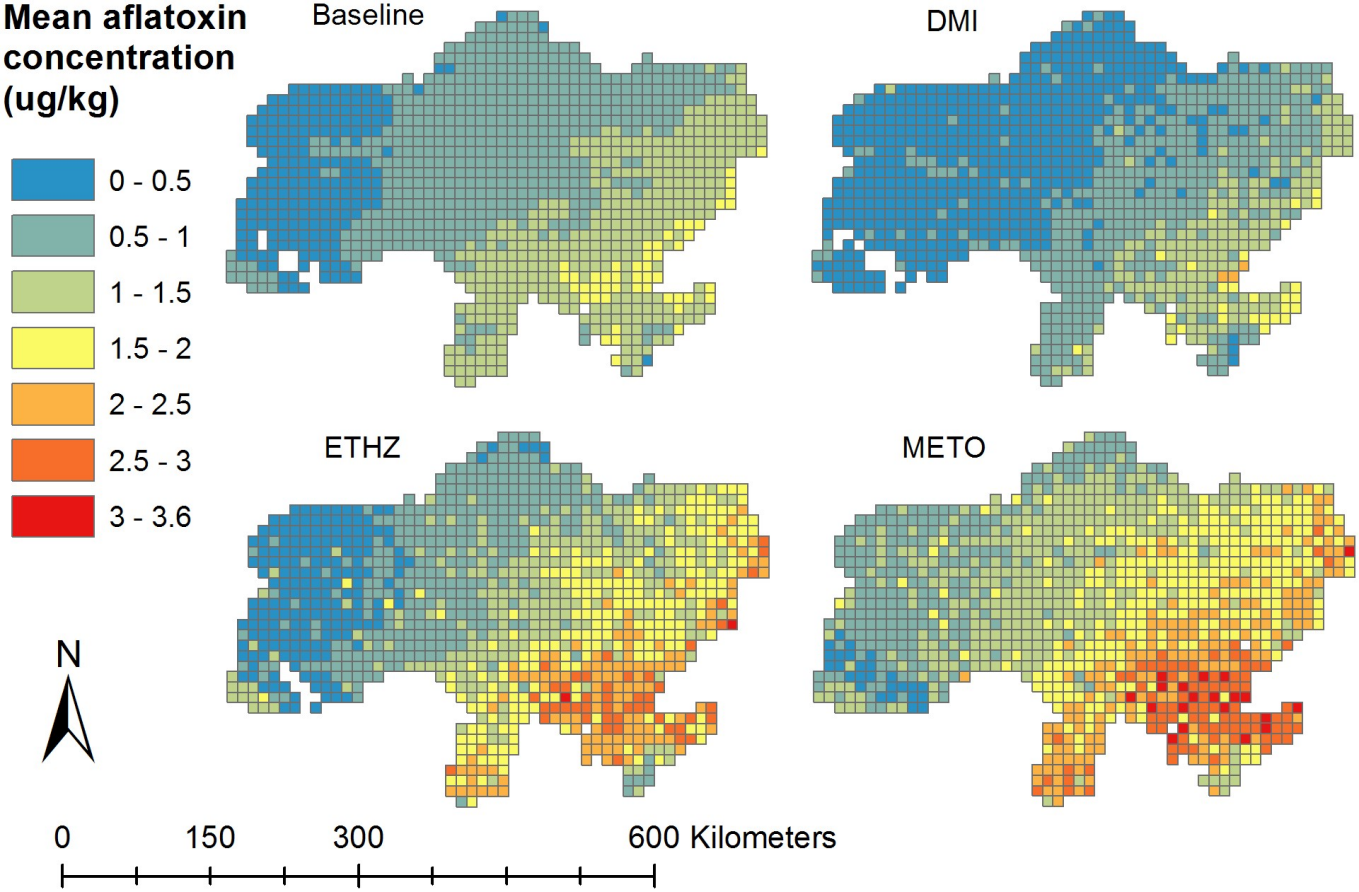
Maps of predicted mean aflatoxin (AfB1) concentration (μg/kg) in maize grown in Ukraine, for baseline conditions and for 2030 using three different climate models (DMI, ETHZ, and METO).

According to our model, maize grown in Ukraine is expected to have a mean AfB1 concentration of 0.8 μg/kg, under baseline weather conditions (Table 3). This mean represents the mean of all simulation runs for all grids. In future, mean AfB1 concentrations of maize grown in Ukraine are expected to decrease (-25% DMI) or increase (+52% ETHZ, +93% METO) for the year 2030. Fig 2 visualizes the predicted mean AfB1 concentrations in maize for the baseline and the different climate models. Local variation in AfB1 concentrations can be observed; highest concentrations are expected in south-east Ukraine (Fig 2).

**Table 3:** Predicted mean and standard deviation of aflatoxin AfB1 concentration (μg/kg) in maize grown in Ukraine under baseline and projected future conditions for three climate models (DMI, ETHZ, METO).

Statistics	Baseline	2030		
		*DMI*	*ETHZ*	*METO*
**Mean**	0.81	0.61	1.22	1.56
**SD**	0.53	0.57	1.07	1.09

### Predicted aflatoxins in Dutch compound feed and milk

Compared to the baseline, the DMI model leads to somewhat lower maximum mean AfM1 concentrations in milk, while the ETHZ model leads to somewhat increased concentrations, and the METO model to up to 50% increased maximum mean concentrations (Table 4). The outcome depends strongly on the transfer equation used, with the EFSA equation (Table 2) being least sensitive to weather changes.

**Table 4:** Maximum of weekly mean AfM1 concentrations (μg/kg) in milk from the whole farm, calculated over 10000 simulations, assuming compound feed contains maize imported from Ukraine.

Transfer equation	Baseline	2030		
		*DMI*	*ETHZ*	*METO*
Masoero et al.2007	9.7E-04	8.4E-04	1.2E-03	1.4E-03
Veldman et al.1992	1.8E-03	1.6E-03	2.2E-03	2.6E-03
Britzi et al. 2013	1.2E-03	1.0E-03	1.4E-03	1.7E-03
Van Eijkeren et al. 2006	1.4E-03	1.2E-03	1.7E-03	2.0E-03
EFSA 2004	1.2E-02	1.2E-02	1.2E-02	1.2E-02

Similarly, for each week, the percentage of the 10000 simulations that exceeded the EC maximum limit of 0.05 μg/kg AfM1 in milk was calculated. The maximum of these weekly percentages are shown in Table 5 for each combination of climate model and transfer equation.

**Table 5:** Maximum weekly percentage of simulations above the EC limit of 0.05 μg/kg AfM1 in milk from the whole farm, assuming compound feed contains maize imported from Ukraine. This percentage is calculated as number of simulations above the threshold/10000 x 100%.

Transfer equation	Baseline	2030		
		*DMI*	*ETHZ*	*METO*
Masoero et al.2007	0.1	0.1	0.1	0.1
Veldman et al.1992	0.4	0.4	0.6	0.6
Britzi et al. 2013	0.1	0.1	0.2	0.2
Van Eijkeren et al. 2006	0.2	0.2	0.3	0.3
EFSA 2004	0.3	0.3	0.4	0.5

The DMI model predicts threshold exceedances to be similar to the baseline. The ETHZ model predicts threshold exceedances slightly higher than the baseline. The strongest increase in the number of times the limit is reached is expected for the METO model. Again the outcome depends strongly on the transfer equation used.

## Discussion

This study aimed to link all available models and data for the case of aflatoxins in the dairy production chain, from the cows’ feed until the food product (milk). Results of this study showed that, given projected climate change, aflatoxin contamination in milk is expected to be comparable to or to increase, relative to the baseline situation. Whether or not an increase is to be expected strongly depends on the climate model and carryover equation used. We used Monte Carlo simulation and the resulting range in outcomes reflect both variability and uncertainty, in both climate modelling and carryover modelling. Uncertainty can be reduced by collecting additional data. E.g., for the carryover model, additional carryover experiments with AfB1 from dairy cows’ feed to AfM1 in milk could be done to reduce the uncertainty related to using the current carryover equations. In the current study, three climate models were used, to represent the range of uncertainty and variability, but only one climate change scenario was considered. Since we focused on the near future, the various climate scenarios do not differ so much, and more difference is expected from the various climate models. In addition, as pragmatic reason, data were available for the current climate scenario data. For looking into the farther future, including multiple climate scenarios as well as multiple climate models would be advisable.

AfB1 contamination of maize grown in Ukraine is generally expected to increase under climate change, particularly with the ETHZ and METO models, but variability increased as well. Mean contamination over all grids in Ukraine and model iterations was higher than in the baseline situation, except for the DMI model, but the standard deviation was higher as well. The DMI model predicts Ukraine to be colder and receive more precipitation than do the ETHZ and METO models (Table 1), so it appears from the models that warmer conditions with less precipitation lead to increased aflatoxin contamination of maize. This is consistent with literature that states the increase in aflatoxins could be due to the expected increase in temperature and relative humidity and/or drought by reduced rainfall since these three factors increase the presence of *A*. *flavus* [34]. The exact effect of the precipitation patterns and relative humidity throughout the year could be investigated in more detail in further research. In their study, Battilani et al. [9, 14] estimated aflatoxin production for a +2 °C and +5 °C increase in temperature considering a period of 100 years. They also observed a general increase in aflatoxin contamination of maize, particularly in Southern Europe, though variation between grids and climate change scenarios was also large.

In the current study, it was not taken into account where (which grid) in Ukraine maize was grown; instead, the predicted aflatoxin levels in maize were averaged over all grid cells in the country (and all years). The reason is that information on the exact origin of maize imported from Ukraine to the Netherlands was not available. However, the current study is a first demonstration of the modelling framework. Since the aflatoxin forecasting model predicts on a 25x25 degree grid, it is possible to select more detailed regions of origin and more specific forecasts can be obtained, likely leading to model outcomes that cover a wider range of values. We focused in this study on AfB1 formation during maize cultivation, up to harvest. AfB1 can also be formed during consecutive transport and storage of the maize, under favourable conditions for *A*. *flavus* to grow and product AfB1. We assumed in this study, transport and storage conditions were well controlled and no additional AfB1 was formed during shipment of the maize to the Netherlands.

This case study focused on a specific safety hazard (AfB1), feed ingredient (maize) and animal (cow) for this workflow. With the chain modelling approach, the transition of this specific toxin throughout the dairy production chain, from “farm-to-fork”, is simulated. The developed framework is flexible so that models for other mycotoxins (e.g. deoxynivalenol) and grain types (e.g. wheat, oats), such as the forecasting model for deoxynivalenol in winter wheat grown in the Netherlands [35], can easily be implemented within this framework. This study used Ukrainian maize as an example for the demonstration purpose. The method presented can be applied for any European country using country specific climate data at JRC agri4cast data portal. More generally, the framework can be extended to available models for other food safety hazards, and crops and/or animal production chains.

The Forecasting model used in this study is based on the AFLA-maize model [10], which is a weather-driven model. This model potentially can be improved and expanded with latest study results on A. flavus growth and AfB1 production [36–38]. These studies provide latest interactions between *Aspergillus* growth and different factors, such as pH, water activities and temperatures. In addition to the weather factors, various other factors can also affect the AfB1 level in maize, such as the resistance level of maize hybrids, fertilizer use, fungicide application, pest pressure and crop management. A forecasting model considering both weather and agronomic factors would be interesting to develop in future research. Studies on other AfB1 producing *Aspergillus* spp., such as *A*. *parasiticus*, can be added in the future studies. Although there are several options to further improve and expand the AFLA-maize model, we chose to simplify it. The reason is that we wanted an approach that can be applied at the European level using (open) available data. Variables such as pH, water activity and local agronomic information are not available with European coverage at this moment.

With increasing globalisation and trade, traceability of food is of continuously growing concern. Food products and ingredients are shipped from one part of the globe to another. Therefore, it has become important for all stakeholders of the food supply chain, from producers, traders and processors up to consumers, to build systems that collect real-time information to trace products from “farm-to-fork”. A traceability system based on blockchains combined with the current modelling framework would enable identification of risky routes in a food production chain. But also as stand-alone modelling framework, scenario analyses can be done, e.g. with climate change scenarios or feeding regimes of cows. Results of such scenario analyses could assist risk managers of private industry and governments in decision making processes.

## Conclusions

This study aimed to investigate the impacts of climate change on AfB1 production in maize and its consequences on AfM1 contamination in dairy cow’s milk, using a full chain modelling approach. Results showed that, given the case study, the scenarios and models used, AfB1 contamination in milk was expected to be comparable or to increase in future climate. For this case study of Ukrainian maize, most of the calculations suggest an increase (up to 50%) of maximum mean AfM1 in milk by 2030, except for DMI model suggesting a decrease. All calculations suggest a stable—with a slightly increase (up to 0.6%)- probability of finding AfM1 in milk above the EC limit of 0.05 μg/kg by 2030. Results mainly depended on the climate model and carryover model used.

The framework infrastructure is flexible so that models for other mycotoxins or food safety hazards and production chains, together with necessary input databases, can easily be included as well. Forecasting models for mycotoxins in other compound feed crops, i.e. wheat, barley, can be added in parallel with the maize forecasting model to study the impact of climate change on mycotoxins in more compound feed ingredients. This study used Eastern European maize as an example for the demonstration purpose. The method presented can be applied for any European country using available country specific climate data. This modelling framework for the first time links datasets and models reported in literature related to AfB1 in maize and related AfM1 in the dairy production chain together to obtain a unique predictive method based on Monte Carlo simulation. Such an integrated approach with scenario analysis provides possibilities for policy makers and risk managers to study the effects of changes in the beginning of the chain on the end product and to adopt appropriate prevention measures.

## Supporting information

S1 File Detailed information on data and models used. (DOC)
