# Supplementary material for: Climate change impacts on aflatoxin B1 in maize and aflatoxin M1 in milk: A case study of maize grown in Eastern Europe and imported to the Netherlands
Source: PLoS One. 2019 Jun 27;14(6):e0218956. doi: 10.1371/journal.pone.0218956 (PMC6597076; doi:10.1371/journal.pone.0218956)
Supplement: S1 File — (DOC) [file pone.0218956.s001.doc]

**Detailed information on data and models used**

“*Climate change impacts on aflatoxins in maize and in the dairy production chain*” by Van der Fels-Klerx et al.

All data and models used in this study as illustrated in Figure 1 of the paper are uploaded on the fig share. Below is a short description of the content of the git repo (Available at: <https://figshare.com/projects/Climate_change_impact_on_Aflatoxin_B1_in_maize_and_Aflatoxin_M1_in_milk/61301>).

Structure of the project with the Git Repo. Each of the models consists of a data folder (input/output) with associated metadata attached to it. The source code associated with each of the models are in the same folder.

Following folders exist:

1. forecastingModels
2. cropPhenologyModels
3. carryOverModels
4. workflows

An overall readme gives the background information on the models and the workflow folder gives an example workflow on how these models can be chained together.

Please note that: TSUM1 and TSUM 2 data are based on the crop phenology model WOFOST (World FOod STudies) developed under the EU Monitoring Agricultural ResourceS (MARS) project. Data used included gridded TSUM1 and TSUM2 for maize grown in Europe, per grid of 25*25 KM. Data is protected. Metadata and some records are shown in de data folder.​

References:

- Van der Fels I, Vermeulen LC, Gavai A, Liu C. Data and source code supporting the publication [Internet]. figshare; 2019.. doi:10.6084/m9.figshare.7863437.v1
- Van der Fels I, Vermeulen LC, Gavai A, Liu C. Fig 2 predicted mean AfB1 concentration in maize [Internet]. figshare; 2019.. doi:10.6084/m9.figshare.7862537.v1
- Van der Fels I, Vermeulen LC, Gavai A, Liu C. Fig 1 setup of the model chain [Internet]. figshare; 2019.. doi:10.6084/m9.figshare.7862561.v1​
